# Complete mitochondrial genome sequence of Awassi-Jo sheep breed (*Ovis aries*) in Jordan

**DOI:** 10.1080/23802359.2021.1906179

**Published:** 2021-03-26

**Authors:** Mohammad H. Brake, Hussein M. Migdadi, Monther T. Sadder, Sami Awabdeh, Khaleel Jawasreh, Wisam Obeidat, Ruba Al Omari, Nizar J. Haddad

**Affiliations:** a Science Faculty, Jerash University, Jerash, Jordan; b National Agricultural Research Center – NARC, Baqa'a, Jordan; c School of Agriculture, The University of Jordan, Amman, Jordan; d Faculty of Agriculture, Jordan University of Science and Technology, Irbid, Jordan

**Keywords:** Evolution, domestication, sheep phylogeny

## Abstract

Using high-throughput sequencing technology, the complete mitochondrial genome of Awassi-Jo breed (*Ovis aries*) was decoded. Mitochondrial genome was 16,617 bp in length. The genome contained 37 genes (13 protein-coding, 22 tRNA, and 2 rRNA) and a control region (D-loop region). The genes were encoded on the H-strand, except for the ND6 gene and 8 tRNA genes, which were encoded on the L-strand. The GC content is 38.9%. Phylogenetic analysis was performed to compare Awassi-Jo with other sheep breeds. The phylogenetic tree showed that Awassi-Jo diverged earlier than related breeds (Turkey, Italy, Germany, and Netherland) with a common ancestor in haplogroup HB. The results revealed the importance of mitochondrial data in studying sheep evolution and domestication.

In Jordan, Awassi-Jo sheep (*Ovis aries*) is the dominant fat tail breed, that has many unique characteristics, including acceptable performance under harsh conditions and preferred meat quality by Mediterranean consumers. Recently, we sequenced the whole genome of male and female Awassi-Jo (Haddad et al. 2020). The whole genomes of both male and female Awassi-Jo were sequenced and the ram genome was published (Haddad et al. 2020). In this study, we assembled the complete mitochondrial genome of Awassi-Jo using next-generation sequencing methods.

The study was approved by national ethical legislation of (Approval Code No. (G/6) 2006). Genomic DNA was extracted using DNA purification kit (Promega, Madison, WI) from whole-blood samples collected from Awassi-Jo sheep. Genomic DNA was prepared and subjected to the Ilumina Hiseq 2500 sequencing system. Library construction of 64 bp pair-end reads and sequencing were carried out using Illumina platform (San Diego, CA).

We performed assembling of mitochondrial genome by CLC Genomics Workbench (Redwood City, CA) by mapping to *O. aries* reference genome (NC_001941), and obtained one contig. Mitochondrial annotations were performed from the above reference genome. The mitochondrial phylogenetic analysis was performed using thirteen previously published mitochondrial genome sequences of *O. aries* from different sheep breeds. Mitochondrial sequences were aligned using multiple alignments (CLC Genomics Workbench). Aligned sequences were used to generate 1000 replicates using PHYLIP (Felsenstein 1989). Bootstrapped data were subjected to the maximum likelihood analysis and a consensus phylogenetic tree was generated.

Mitochondrial genome was 16,617 bp in length. The genome contained 37 genes (13 protein-coding, 22 tRNA, and 2 rRNA) and a control region (D-loop region). The genes were encoded on the H-strand, except for the ND6 gene and 8 tRNA genes, which were encoded on the L-strand. The GC content is 38.9%. A total of 42 polymorphic loci were found in Awassi-Jo mitochondrial genome when compared with the *O. aries* reference (NC_001941), they included 3 insertions, 2 deletions, and 37 single nucleotide polymorphisms (SNPs). Most of them were located in intergenic space, while two insertions were found in 12S rRNA and 16S rRNA genes. On the other hand, a total of 151 polymorphic loci were found in Awassi-Jo mitochondrial genome when compared with the related Awassi breed (HM236182), which included 1 insertion, 4 deletions, and 146 SNPs. The phylogenetic tree showed that the available breed's mitochondrial sequences are clustered in two main clades (Figure 1). The first included two subclades covering haplogroups HC and HE based on *O. aries* haplogroup classification (Meadows et al. 2011). The second clade included three subclades covering HA, AB, and HD. Awassi-Jo breed is grouped with breeds from Turkey, Italy, Germany, and Netherland in the HB haplogroup based. However, Awassi-Jo was shown to diverge earlier than related HB breeds. This agrees with previous citation suggesting that sheep were first domesticated in the Fertile Crescent region including Jordan and spread out into the world (Chessa et al. 2009; Haddad et al. 2020).

**Figure 1.**
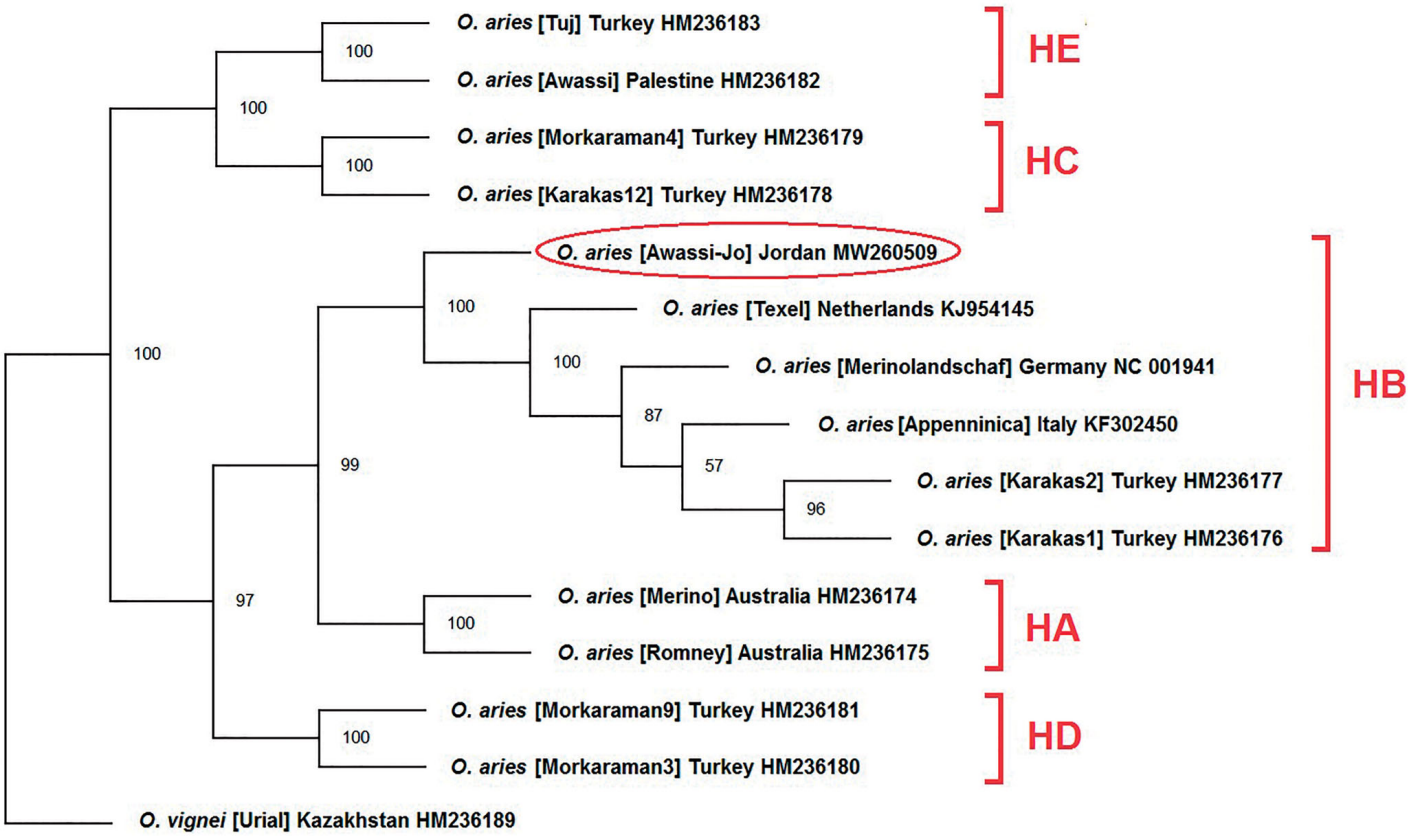
Mitochondrial genome maximum likelihood phylogenetic tree of Awassi-Jo (*Ovis aries*) along with other breeds. Bootstrap values are given on each branch (1000 replicate). *O. vignei* was used as out-groups. Major clades were assigned to corresponding haplogroups (HA, HB, HC, HD, and HE) based on Meadows et al. (2011) classification.

## Data Availability

The data that support the findings of this study are openly available in Genbank (NCBI, 2021) with accession number MW260509 and raw data with accession number SRX7765381. A voucher specimen is kept at NARC, Jordan.
